# Dietary Intake of Calcium and Magnesium in Relation to Severe Headache or Migraine

**DOI:** 10.3389/fnut.2021.653765

**Published:** 2021-03-05

**Authors:** Shu-Han Meng, Ming-Xue Wang, Li-Xin Kang, Jin-Ming Fu, Hai-Bo Zhou, Xin Li, Xia Li, Xue-Ting Li, Ya-Shuang Zhao

**Affiliations:** Department of Epidemiology, College of Public Health, Harbin Medical University, Harbin, China

**Keywords:** migraine, dietary calcium, dietary magnesium, NHANES, cross-sectional study

## Abstract

**Background:** Migraine is a common neurological disorder and is affected by nutrients. Calcium and magnesium are essential minerals that play an important role in nerve function. So we investigated the association between dietary calcium and magnesium and migraine.

**Methods:** We extracted 10,798 adults from the National Health and Nutrition Examination Surveys (NHANES) of America in 1999 to 2004. We classified patients who reported having severe headache or migraine as having possible migraine. Multivariable logistic regression and restricted cubic spline regression were conducted to determine the association between dietary calcium and magnesium and migraine.

**Results:** We found that the adjusted ORs of the association between dietary calcium and magnesium and migraine for comparing the highest quintile intake with the lowest quintile intake were 0.77 (95% CI: 0.63–0.93, *P* = 0.008) and 0.69 (95% CI: 0.55–0.86, *P* = 0.001), respectively. For women, the adjusted ORs of dietary calcium and magnesium were 0.72 (95% CI: 0.56–0.93, *P* = 0.009) and 0.62 (95% CI: 0.47–0.83, *P* = 0.001), respectively. For men, the adjusted OR was 0.71 (95% CI: 0.52–0.97, *P* = 0.028) comparing the highest and the lowest quintile of calcium intake, but there was no statistically significant association between dietary magnesium intake and migraine. Joint analyses showed that the OR in the high-calcium and high-magnesium group was 0.74 (95% CI: 0.60–0.92, *P* = 0.006) compared with the low-calcium and low-magnesium group in women.

**Conclusions:** High dietary intake of calcium and magnesium, independently or in combination, were inversely associated with migraine in women. For men, high dietary calcium was negatively related to migraine, but magnesium was not associated with migraine.

## Introduction

Migraine is a common neurological disease and is associated with severe socioeconomic burdens (1). Migraine is influenced by many factors, such as genetic, environment, behavior, and nutrition, etc. Previous studies have shown that natural compounds play an important role in human health (2–6), while other studies have shown that diet and nutrients are closely related to migraine (7–9). Therefore, identifying these factors, especially modifiable ones like nutrition, may help prevent and treat migraine.

Calcium and magnesium are essential minerals in the human body, and they are intimately related to each other and collectively influence several physiological functions, such as normal cellular physiology, signal transduction, and neurotransmitter release (10, 11). Calcium and magnesium need to be obtained from the outside and cannot be manufactured by the human body. Magnesium could inhibit neuronal overexcitation and vasospasm, reduce the formation of inflammatory substances, and improve mitochondrial oxidative phosphorylation and serotonin receptor transmission (12–14). Previous studies have shown that migraineurs have lower serum magnesium levels than the normal population, and magnesium deficiency is strongly associated with migraine (15, 16). Intravenous magnesium (17–19), oral magnesium preparation (20, 21), or magnesium supplements (22, 23) have been found to be effective against migraine.

Few studies have reported an association between calcium and migraine. However, studies have shown that vitamin D deficiency is associated with migraine (24, 25). Several studies have also indicated that vitamin D supplements could improve migraine (26, 27). It is well-known that promoting calcium absorption is an important physiological function of vitamin D (28). Besides, two case reports showed that two menstrual migraineurs had a reduction in the frequency and duration of migraine attacks after receiving a combination of vitamin D and calcium supplements (29). Therefore, we believe that calcium may play an important role in migraine.

Currently, most studies on calcium and magnesium for migraine prevention are limited to drugs and supplements. However, most of these drugs and supplements have diverse limitations and side effects, such as gastrointestinal discomfort, nephrolithiasis, and cardiovascular disease (12, 30, 31). In recent years, the effects of dietary therapy on migraine have received increased research attention. Many nutrients have also been demonstrated to be protective against migraine, such as riboflavin, coenzymes Q10, vitamin B12, and omega-3 fatty acid, etc. (13, 32, 33). However, to our knowledge, studies that directly examine the association between dietary calcium and magnesium and migraine are rare.

Therefore, the purpose of this cross-sectional study was to investigate the association between dietary calcium and magnesium intake, individually or in combination, and migraine using 6 years of NHANES data (1999–2004). We also explored whether the relationship would differ by sex. We hypothesized that higher dietary magnesium and calcium intake, independently or in combination, would be inversely associated with migraine.

## Methods

### Study Populations

This cross-sectional study used data from the National Health and Nutrition Examination Survey (NHANES), administered by the Centers for Disease Control and Prevention (CDC) (34). The NHANES data includes a series of cross-sectional, stratified, multistage probability surveys of the civilian, noninstitutionalized populations in the US. The NHANES provides information on demographics, physical examinations, laboratory tests, diet surveys, and other health-related questions. Data were collected by the National Center for Health Statistics (NCHS) and approved by their ethical review board (35). The NHANES obtained the informed consent of all participants. Data from NHANES are publicly available and can be downloaded on the NHANES website (http://www.cdc.gov/nchs/nhanes.htm).

A total of 15,332 participants, aged 20 years or older, were surveyed for severe headaches or migraines from NHANES 1999–2004. We excluded pregnant women (*n* = 833) and participants with basic information missing such as demographics (*n* = 526), diet surveys (*n* = 1,710), physical examinations (*n* = 282), laboratory tests (*n* = 567), and other health-related surveys (*n* = 616). Finally, 10,798 adults (5,526 men and 5,272 women) were included in our study.

### Headache Assessment

Severe headache or migraine were assessed by self-report in the miscellaneous pain section of the NHANES questionnaire. We classified participants who answered “yes” as severe headache sufferers and migraineurs: “Have you had a severe headache or migraine in the last 3 months?” We have reason to assume that most participants with severe headaches suffer from migraines. The results of the American Migraine Prevalence and Prevention (AMPP) study support our assumption. The study showed that 17.4% of participants reported “severe headache,” of which 11.8% met the International Headache Disorder type II (ICHD-II) criteria for migraine, 4.6% met the criteria for “possible migraine,” and only 1% were classified as “other severe headache” (36). Therefore, we classified patients who reported having severe headache or migraine as having possible migraine.

### Dietary Assessment

The NHANES dietary survey recorded information on the type and amount of all foods and beverages consumed by participants within 24 h. The 24-h dietary recalls were collected using the computer-assisted dietary interview (CADI) system from 1999 to 2002, while they were collected using the United States Department of Agriculture's (USDA) Automated Multiple Pass Method (AMPM) from 2003 to 2004. CADI and AMPM collected detailed information about each individual's foods and beverages and converted it into various nutrients (37). Detailed dietary survey methods were provided in the NHANES Dietary Interviewers Procedure Manuals (38). Despite the inherent limitations of 24-h dietary recalls in terms of reliability and effectiveness of nutritional assessments, studies suggest that 24-h dietary recalls may provide more details about the type and quantity of food than food frequency surveys (39, 40).

### Covariates Assessment

The potential covariates included demographic characteristics (age, sex, race/ethnicity, education, and marital status), lifestyles (drinking and smoking), diet assessments (energy intake, protein intake, and carbohydrate intake), calcium and magnesium supplements, vitamin D supplements, physical examinations and laboratory tests (body mass index (BMI), total cholesterol, and C reactive protein), disease history including hypertension (defined as systolic blood pressure ≥140 mm Hg or diastolic blood pressure ≥90 mm Hg and obtained from self-report and took anti-hypertensive medications), diabetes (obtained from self-report and took anti-diabetic medications), stroke and coronary heart disease (obtained from self-report).

### Statistical Analyses

Statistical analyses were conducted using R software (version 3.2.5). All analyses in this study were based on weighted estimates of sample weights provided by NHANES (41). Continuous and frequency variables were expressed as mean ± standard deviation and percentage, and their differences between headache and non-headache individuals were evaluated by Student *t*-test and Chi-square test, respectively. We divided participants into five quintiles based on dietary calcium and magnesium intake. A multivariable logistic regression was used to explore the association between dietary calcium and magnesium intake and migraine, and data were expressed as OR (95% CI).

Restricted cubic spline (RCS) regression (42) was performed to explore the non-linear relationship between dietary calcium and magnesium and migraine. Calcium and magnesium were included in the model as continuous variables. We choose the median of dietary calcium or magnesium, which is not affected by the extreme value of the distribution sequence and has representativeness to the distribution sequence, as the reference value. The knots were placed at the 5th, 25th, 50th, 75th, and 95th percentiles. Two-sided *P* < 0.05 were considered statistically significant. *P*-values were adjusted for multiple comparisons using the Bonferroni correction.

## Results

Table 1 shows the basic characteristics of the 10,798 participants who had complete information from the NHANES 1999–2004 data. Of these participants, 2,123 (19.7%) had migraine. Compared to non-headache, headache were more likely to be younger, female, non-Hispanic Black, former drinking, current smoking, have lower education level, lower prevalence of hypertension, higher BMI, higher carbohydrate intake, lower calcium and magnesium intake, and lower vitamin D supplement intake. The average daily intake of calcium and magnesium was significantly lower than their respective recommended dietary allowances (RDAs), as shown in Table 2.

**Table 1:** Characteristics of participants with or without headache.

**Characteristic**	**Non-headache**	**Headache**	***P*_**value**_**
Participants, No. (%)	8,675 (80.3)	2,123 (19.7)	
Age, Mean (SD), years	52.77 (18.96)	45.07 (15.95)	<0.001^a^
**Sex**			
Male	4,759 (54.9)	767 (36.1)	<0.001^b^
Female	3,916 (45.1)	1,356 (63.9)	
**Race/ethnicity No. (%)**			
Non-Hispanic white	4,702 (54.2)	1,028 (48.4)	<0.001^b^
Non-Hispanic black	1,547 (17.8)	436 (20.5)	
Mexican American	1,791 (20.6)	469 (22.1)	
Others	635 (7.3)	190 (8.9)	
**Marital status No. (%)**			
Married or living with partner	5,413 (62.4)	1,275 (60.1)	0.046^b^
Living alone	3,263 (37.6)	848 (39.9)	
**Education level No. (%), years**			
<High School	2,588 (29.8)	727 (34.2)	<0.001^b^
High school or GED	2,069 (23.9)	530 (25.0)	
>High School	4,018 (46.3)	866 (40.8)	
**Drinking No. (%)**			
Never	1,201 (13.8)	328 (15.4)	<0.001^b^
Current	6,099 (70.3)	1,377 (64.9)	
Former	1,375 (15.9)	418 (19.7)	
**Smoking No. (%)**			
Never	4,302 (49.6)	1,071 (50.4)	<0.001^b^
Current	1,824 (21.0)	603 (28.4)	
Former	2,549 (29.4)	449 (21.1)	
Hypertension No. (%)	2,692 (31.0)	533 (25.1)	<0.001^b^
Diabetes No. (%)	918 (10.6)	205 (9.7)	0.210^b^
Stroke No. (%)	356 (4.1)	78 (3.7)	0.575^b^
Coronary heart disease No. (%)	393 (4.5)	85 (4.0)	0.403^b^
BMI, Mean (SD), kg/m^2^	27.64 (5.69)	28.26 (6.56)	<0.001^a^
Energy (kcal/day)	2,112.96 (1,003.88)	2,116.15 (1,087.87)	0.897^a^
Protein intake (g/day)	79.94 (41.48)	77.98 (45.63)	0.056^a^
Carbohydrate intake (g/day)	259.45 (130.50)	268.38 (145.26)	0.006^a^
Total cholesterol (mmol/L)	5.26 (1.10)	5.19 (1.07)	0.027^a^
C reactive protein (mg/dL)	0.23 (0.14)	0.27 (0.10)	0.102^a^
Calcium (mg/day)	713.49 (457.29)	676.72 (426.52)	0.001^a^
Magnesium (mg/day)	256.48 (142.94)	237.84 (133.28)	<0.001^a^
Calcium supplement No. (%)	1,780 (20.5)	428 (20.2)	0.713^b^
Magnesium supplement No. (%)	208 (2.4)	43 (2.0)	0.308^b^
Vitamin D supplement No. (%)	1,932 (22.3)	375 (17.7)	<0.001^b^

*BMI, body mass index (calculated as weight in kilograms divided by height in meters squared); SD, standard deviation*.

*Continuous and frequency variables were expressed as the mean (SD) and percentage*.

a *Comparing the two groups with Student t-test*.

b *Comparing the two groups with Chi-square test*.

**Table 2:** Dietary calcium and magnesium intake among US adults (≥20 years) in NHANES 1999–2004.

**Age (years)**	**RDAs for calcium (mg/day)**	**Calcium intake (mg/day)**	***P*-value**
20–50^a^	1,000	643.86 (402.73)	<0.001
51–85^a^	1,200	579.28 (345.46)	<0.001
20–50^b^	1,000	852.50 (527.06)	<0.001
51–85^b^	1,200	675.49 (403.29)	<0.001
**Age (years)**	**RDAs for magnesium (mg/day)**	**Magnesium intake (mg/day)**	***P*****-value**
20–30^a^	310	216.36 (101.20)	<0.001
31–85^a^	320	218.03 (105.11)	<0.001
20–30^b^	400	300.72 (153.44)	<0.001
31–85^b^	420	289.41 (156.73)	<0.001

a *Female; ^b^Male*.

Table 3 shows a significant inverse association between dietary calcium and magnesium intake and migraine after adjusting for the possible confounders. The ORs (95% CI) for the association between dietary calcium intake and migraine were 0.85 (0.73–0.99) and 0.77 (0.63–0.93), comparing the Q2 (378.01–571.00 mg/day) and Q5 (≥1,149.01 mg/day) quintile of calcium with the lowest quintile (≤378.00 mg/day), respectively. The ORs (95% CI) of Q2 (161.01–217.00 mg/day), Q3 (217.01–282.00 mg/day), Q4 (282.01–371.00 mg/day), and Q5 (≥371.01 mg/day) quintile of magnesium were 0.81 (0.69–0.95), 0.83 (0.70–0.98), 0.78 (0.65–0.94), and 0.69 (0.55–0.86) compared with Q1 (<161.00 mg/day).

**Table 3:** Association between dietary calcium and magnesium and headache.

		**OR (95% CI)**					
	**No**.	**Crude**	***P-*value**	**Model 1^a^**	***P-*value**	**Model 2^b^**	***P-*value**
**Dietary calcium (mg/day)**							
Q1 (≤378.00)	2,160	1 (Ref.)		1 (Ref.)		1 (Ref.)	
Q2 (378.01–571.00)	2,163	0.84 (0.72–0.97)	0.019	0.86 (0.73–1.00)	0.054	0.85 (0.73–0.99)	0.044
Q3 (571.01–800.00)	2,162	0.83 (0.72–0.96)	0.014	0.86 (0.73–1.01)	0.074	0.87 (0.74–1.02)	0.094
Q4 (800.01–1,149.00)	2,158	0.85 (0.73–0.98)	0.026	0.85 (0.72–1.01)	0.063	0.87 (0.73–1.03)	0.097
Q5 (≥1,149.01)	2,155	0.77 (0.67–0.90)	0.001	0.77 (0.63–0.93)	0.007	0.77 (0.63–0.93)	0.008
***P***_**trend**_		0.012		0.102		0.098	
**Dietary magnesium (mg/day)**							
Q1 (≤161.00)	2,160	1 (Ref.)		1 (Ref.)		1 (Ref.)	
Q2 (161.01–217.00)	2,173	0.78 (0.67–0.90)	0.001	0.81 (0.69–0.94)	0.007	0.81 (0.69–0.95)	0.007
Q3 (217.01–282.00)	2,168	0.76 (0.65–0.87)	<0.001	0.82 (0.70–0.97)	0.019	0.83 (0.70–0.98)	0.026
Q4 (282.01–371.00)	2,148	0.72 (0.62–0.83)	<0.001	0.78 (0.65–0.93)	0.007	0.78 (0.65–0.94)	0.008
Q5 (≥371.01)	2,149	0.65 (0.56–0.75)	<0.001	0.69 (0.55–0.86)	0.001	0.69 (0.55–0.86)	0.001
***P***_**trend**_		<0.001		0.013		0.015	

*Q, quintile; Ref, reference*.

a *Model 1 was adjusted for age, sex, and race/ethnicity, smoking, drinking, marital, education level, BMI, energy intake, protein intake, and carbohydrate intake, calcium supplement, magnesium supplement, and vitamin D supplement*.

b *Model 2 was adjusted for all variables in Model 1 as well as baseline diseases (hypertension, diabetes, stroke, and coronary heart disease) and clinical characteristics (C reactive protein, total cholesterol)*.

**Figure 1:**
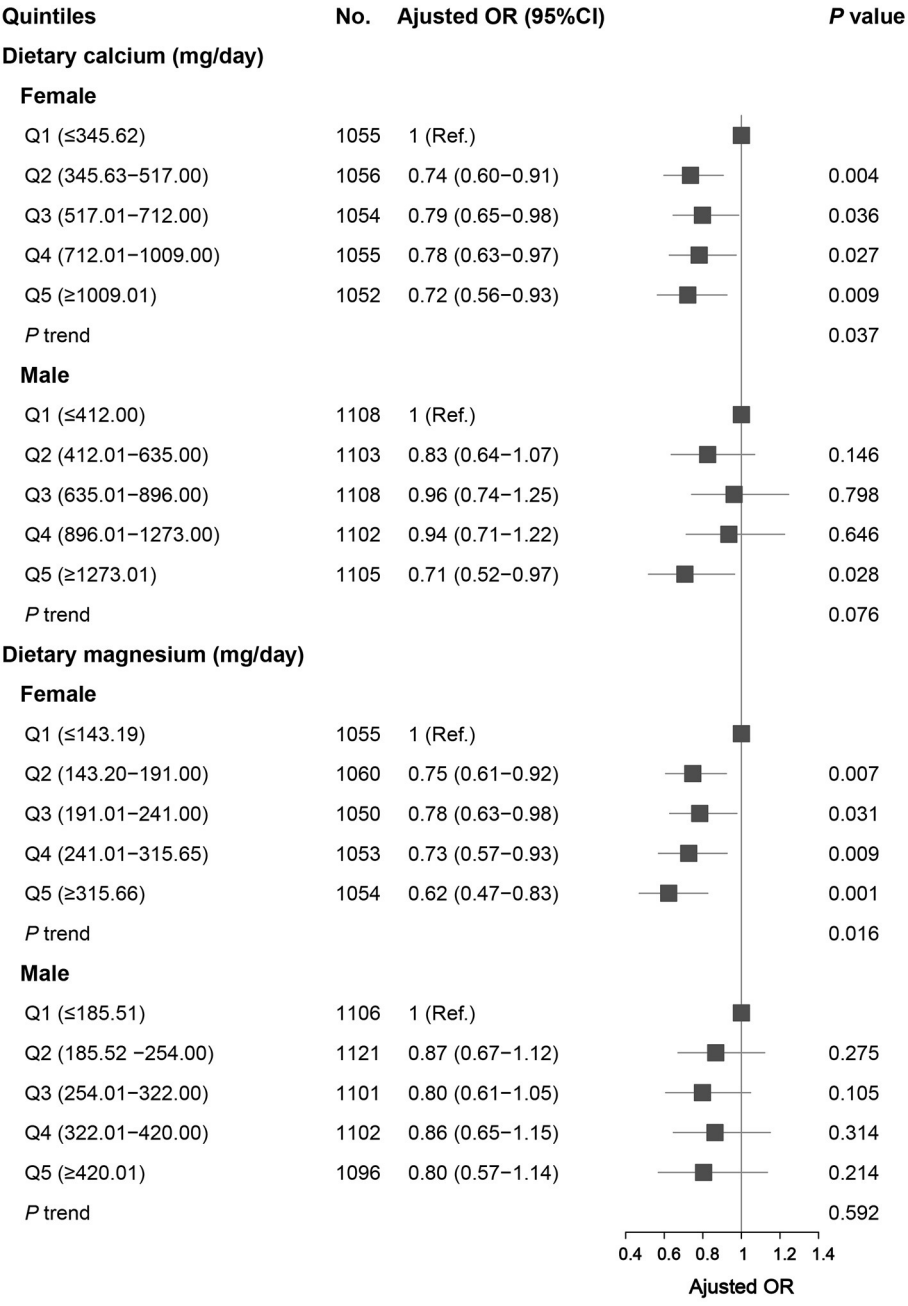
Association between dietary calcium and magnesium intake and headache in different sex groups. Data were presented as OR (95% CI). Limit lines indicate 95% CI. ORs were adjusted for age, race/ethnicity, smoking, drinking, marital, education level, BMI, energy intake, protein intake, carbohydrate intake, calcium supplement, magnesium supplement, vitamin D supplement, hypertension, diabetes, stroke, coronary heart disease, C reactive protein, and total cholesterol.

After stratifying by sex, the association between the highest and the lowest quintile of calcium intake and migraine remained statistically significant in women and men. For dietary magnesium, the OR (95% CI) was 0.62 (0.47–0.83), comparing the highest (≥315.66 mg/d) and the lowest quintile (≤143.19 mg/d) of magnesium intake in women. However, there was no statistically significant association between dietary magnesium intake and migraine in men (Figure 1).

We also examined the potential correlation and joint association between calcium and magnesium. We found that calcium and magnesium were highly correlated (*r* = 0.631; *P* < 0.001) (data are not shown). We divided participants into four groups based on the quintile intake of calcium and magnesium, after adjusting for potential confounders, joint analyses showed that the OR (95% CI) of migraine in the high-calcium and high-magnesium group was 0.77 (0.64–0.91) compared with the low-calcium and low-magnesium group. After stratifying by sex, the joint association of dietary calcium and magnesium remained significant in women. However, the joint association was not significant in men (Table 4).

**Table 4:** Joint association of dietary calcium and magnesium on headache.

	**No**.	**OR (95% CI)^a^**	***P-*value**	**No**.	**OR (95% CI)**	***P-*value**
**All individuals**		**Low Ca intake^b^**			**High Ca intake^c^**	
Low Mg intake^b^	1,210	1 (Ref.)		950	0.93 (0.76, 1.15)	0.501
High Mg intake^c^	950	0.87 (0.70, 1.09)	0.224	7,688	0.77 (0.64, 0.91)	0.003
**Women**		**Low Ca intake^b^**			**High Ca intake^c^**	
Low Mg intake^b^	765	1 (Ref.)		389	0.93 (0.70, 1.24)	0.617
High Mg intake^c^	298	0.87 (0.61, 1.18)	0.154	3,820	0.74 (0.60, 0.92)	0.006
**Men**		**Low Ca intake^b^**			**High Ca intake^c^**	
Low Mg intake^b^	445	1 (Ref.)		173	1.00 (0.60, 1.68)	0.999
High Mg intake^c^	239	0.73 (0.44, 1.22)	0.235	4,669	0.98 (0.72, 1.36)	0.940

*Ca, calcium; Mg, magnesium*.

a *Adjusted for age, sex, race/ethnicity, smoking, drinking, marital, education level, BMI, energy intake, protein intake, carbohydrate intake, calcium supplement, magnesium supplement, vitamin D supplement, hypertension, diabetes, stroke, coronary heart disease, C reactive protein, and total cholesterol*.

b *The lowest quintile intake of calcium or magnesium (Q1)*.

c *The second to fifth quintiles intake of calcium or magnesium (Q2–Q5)*.

**Figure 2:**
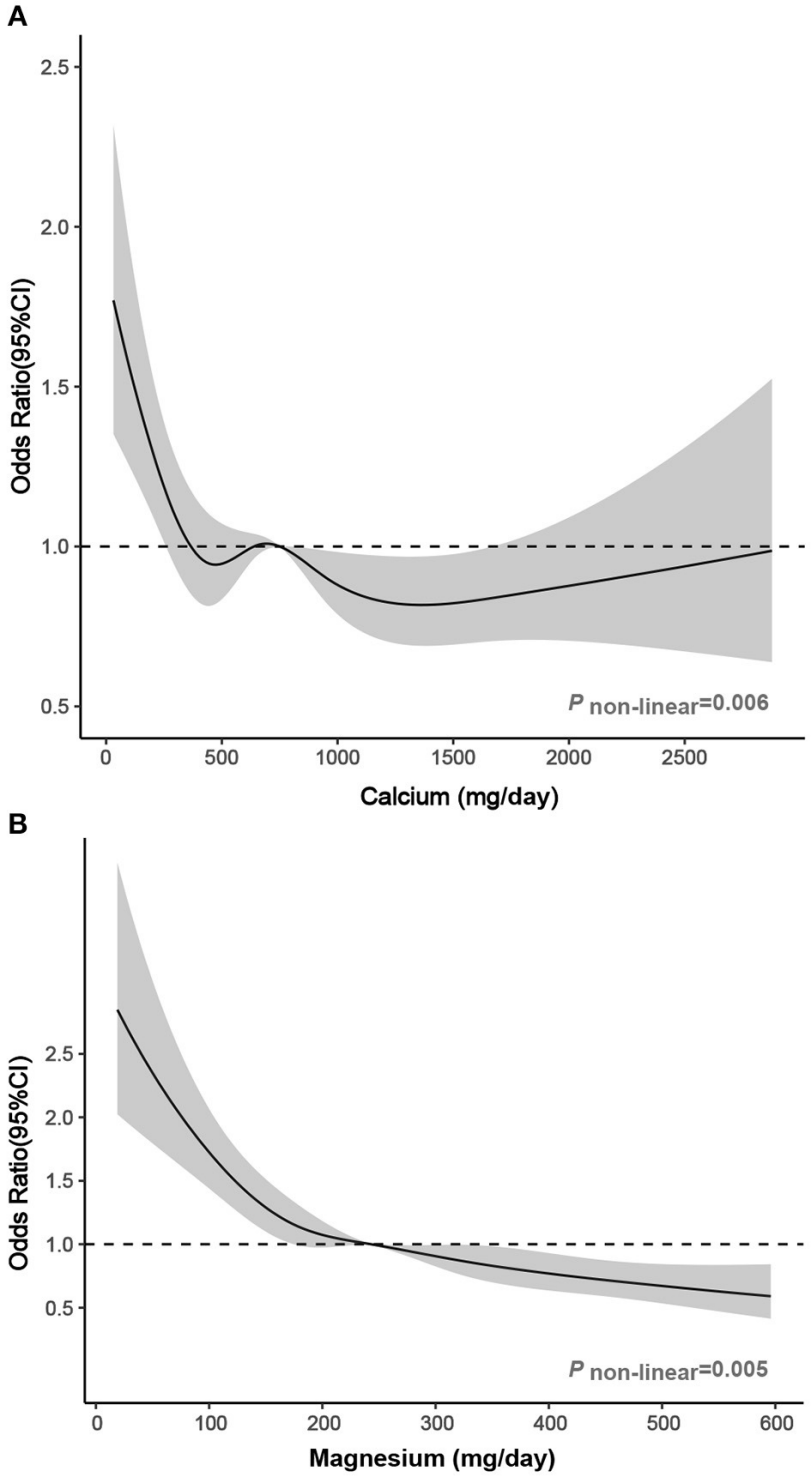
Association between dietary **(A)** calcium and **(B)** magnesium intake and headache in RCS. The model was adjusted for age, sex, race/ethnicity, smoking, drinking, marital, education level, BMI, energy intake, protein intake, carbohydrate intake, calcium supplement, magnesium supplement, vitamin D supplement, hypertension, diabetes, stroke, coronary heart disease, C reactive protein, and total cholesterol. Solid line, OR; shade, 95% CI.

In RCS, we observed a non-linear relationship between calcium and magnesium (continuously measured) and migraine. Significantly decreased ORs (<1.00) were observed when calcium intake ranged from 670 to 1,700 mg/day. The ORs decreased with increasing dietary magnesium levels and were significantly lower than 1.00 when dietary magnesium intake was higher than 243 mg/day (Figure 2).

## Discussion

In this large cross-sectional study, we observed an inverse association between dietary calcium and magnesium and migraine, independent of significant confounders. Subgroup analysis revealed that calcium and magnesium remained inversely associated with migraine in women, respectively. For men, only high dietary calcium intake was negatively related to migraine, but there was no statistically significant association between dietary magnesium intake and migraine. Furthermore, in analyzing the combined effects of calcium and magnesium, we found that high-calcium and high-magnesium intake was inversely associated with migraine in women, consistent with our study of the independent effects of calcium and magnesium.

Previous studies may provide some indirect evidence to support our results. Some studies have found that people with migraine have lower magnesium levels in serum and brain tissue than the healthy population (15, 16). Intravenous magnesium has been found to be effective in migraine (17–19). Some randomized controlled trials have shown that oral magnesium preparations or supplements could reduce the severity and frequency of migraine (20–23). However, few studies have reported the effects of calcium on migraine. More attention has been paid to the effects of Vitamin D on migraine. Studies have shown that oral vitamin D supplements can relieve migraine (26, 27). Two case reports indicated that the frequency and duration of migraine attacks were reduced after taking vitamin D and calcium supplements in menstrual migraineurs (29). The main physiological function of vitamin D is to promote calcium absorption (28). Therefore, we believe that calcium also plays a vital role in migraine prevention and treatment.

Although these studies suggest magnesium and calcium supplements could alleviate migraine, these supplements have some side effects. Studies have shown that calcium and magnesium supplements can cause gastrointestinal symptoms such as abdominal pain, nausea, and diarrhea (12). In addition, calcium supplements can increase the risk of nephrolithiasis and cardiovascular disease (30, 31). There is a growing interest in studying the role of natural compounds in human health (2–6), and many studies have shown that diet and nutrients play an important role in the prevention and treatment of migraine (7–9). Dietary calcium and magnesium are safer and more convenient than supplements and should be recommended as a priority. Our results showed that high dietary calcium and magnesium intakes were negatively related to migraine. However, we also found that the average intake of daily calcium and magnesium of U.S. adults was lower than their RDAs. Therefore, American adults should raise their awareness of RDA for calcium and magnesium and increase dietary calcium and magnesium intake, which may become an effective way to prevent migraine. Green leafy vegetables, fruits, whole grains, nuts, seeds, fish, and beans are rich in magnesium (43, 44). Dairy foods, soy products, grains, seafood, and fortified foods are good dietary sources of calcium (44).

The biological mechanisms between dietary calcium and magnesium intake and migraine were not fully understood but may be related to the following mechanisms. Magnesium may prevent migraine by inhibiting neuronal overexcitation, counteracting vasospasm, and reducing the formation of inflammatory mediators, as well as improving mitochondrial oxidative phosphorylation, serotonin receptor transmission, and the NO system (12–14). Calcium and magnesium, which are involved in the synthesis and release of various neurotransmitters and inflammatory mediators, may work together to help the nervous system function properly and relieve nerve tension (10, 11). Our results support the positive role of magnesium and calcium in migraine, the only difference being that we did not find an effect of magnesium on migraine in men. We speculate that this might be due to significantly higher ionized magnesium levels in the muscles of men than in women (45). Therefore, the effect of dietary magnesium intake on actual magnesium levels may be attenuated in men. Further prospective longitudinal studies and mechanistic studies are needed to elucidate the association between dietary calcium and magnesium intake and migraine.

Our study has several strengths. To our knowledge, this is the first and largest nationally representative sample study to evaluate the association between dietary calcium and magnesium and migraine in US adults. In addition, we assessed the dose-response effects of dietary calcium and magnesium on migraine and provided more practical recommendations. There were some limitations. First, our results may not be appropriate for generalizing the world population since the study participants were all Americans. Second, migraine was not classified according to the International Headache Classification criteria. However, the AMPP study indicated that most patients with severe headache met the classification criteria for migraine, so we believe that the diagnosis of migraine is relatively accurate. In future studies, defining diagnostic criteria for headache will help to better identify people who benefit from dietary calcium and magnesium. Third, although we have controlled for vitamin D supplements, we lack information on dietary vitamin D. Future prospective studies should consider the effect of dietary vitamin D to further investigate the association between calcium and migraine. Finally, our study is a cross-sectional study, which cannot make a causal inference, and further prospective longitudinal studies are needed.

## Conclusions

Our study found that high dietary intake of calcium and magnesium, independently or in combination, were inversely associated with migraine in women. For men, high dietary calcium intake was negatively related to migraine. People should pay more attention to dietary calcium and magnesium, which may be an effective way to prevent migraine. Although changing dietary habits requires great motivation, such efforts may be effective and rewarding for migraine.

## Data Availability Statement

The datasets presented in this study can be found in online repositories. The names of the repository/repositories and accession number(s) can be found here: http://www.cdc.gov/nchs/nhanes.htm.

## Ethics Statement

The studies involving human participants were reviewed and approved by the National Center for Health Statistics Institutional Review Board. The patients/participants provided their written informed consent to participate in this study.

## Author Contributions

S-HM, M-XW, L-XK, and Y-SZ contributed to the conception and design of the study. All authors contributed to analysis and interpreted data. S-HM and Y-SZ contributed to drafting the text. All authors contributed to the article and approved the submitted version.

## Conflict of Interest

The authors declare that the research was conducted in the absence of any commercial or financial relationships that could be construed as a potential conflict of interest.
